# Bipolar and major depressive disorders: associations with serum zonulin levels and rs2070937 polymorphism

**DOI:** 10.1186/s12888-025-07548-y

**Published:** 2025-11-13

**Authors:** Ozgur Baykan, Furkan Akbas, Ayla Solmaz Avcikurt, Hayriye Baykan

**Affiliations:** 1 Department of Medical Biochemistry, Faculty of Medicine, Balıkesir University, Balıkesir, Turkey; 2 Department of Psychiatry, Faculty of Medicine, Balıkesir University, Balıkesir, Turkey; 3 Department of Medical Genetics, Faculty of Medicine, Balıkesir University, Balıkesir, Turkey

**Keywords:** Zonulin, Bipolar disorder, Major depressive disorder, Gut–brain axis, rs2070937, Genetic polymorphism

## Abstract

**Background:**

The underlying pathophysiology of bipolar disorder and major depressive disorder is not fully understood. Inflammation is increasingly recognized as a contributing factor. The gut plays a central role in this process. Increased intestinal permeability, which promotes inflammation, is regulated by tight junctions and influenced by the zonulin protein. This mechanism has been linked to both inflammatory and psychiatric disorders. This study investigates differences in serum zonulin levels and the rs2070937 genetic polymorphism among patients with bipolar disorder, major depressive disorder, and healthy controls.

**Methods:**

A total of 47 patients with bipolar disorder, 56 patients with major depressive disorder, and 51 healthy controls were enrolled. Manic and depressive symptom severity were assessed with the Young Mania Rating Scale and the Hamilton Depression Rating Scale. Venous blood samples were collected from all participants to evaluate serum zonulin levels and to perform genotyping of the rs2070937 polymorphism.

**Results:**

Serum zonulin levels were significantly higher in patients with bipolar disorder compared to healthy controls (p_adj_ = 0.012), and in patients with major depressive disorder compared to healthy controls (p_adj_ < 0.001). No significant differences were observed between the bipolar disorder and major depressive disorder groups. Analysis of rs2070937 genotypes (AA, AG, GG) revealed no significant differences in serum zonulin levels within genotype groups. Furthermore, the distribution of genotypes did not differ significantly among the bipolar disorder, major depressive disorder, and healthy control groups.

**Conclusions:**

Our findings suggest that increased gut permeability may contribute to the pathophysiology of bipolar disorder and major depressive disorder. Recognizing the role of the gut–brain axis in mood disorders could facilitate earlier screening and support the development of personalized treatment approaches.

## Background

Mood disorders are a major global cause of disability, morbidity, and mortality [1]. Among them, major depressive disorder (MDD) and bipolar disorder (BD) are the most prevalent, characterized by emotional and cognitive impairments that substantially limit daily functioning and reduce quality of life [2, 3]. The global prevalence is estimated at approximately 3.4% for MDD and 0.5% for BD [1].

BD is defined by manic episodes, which are distinct periods of persistently elevated, irritable, or expansive mood accompanied by abnormally increased activity or energy [4]. MDD is characterized by a persistent depressed mood, loss of interest or pleasure, feelings of worthlessness or excessive guilt, low energy, cognitive impairment, disturbances in sleep or appetite, psychomotor changes, and suicidal thoughts [4]. Despite their well-defined clinical features, the etiology and underlying mechanisms of BD and MDD remain incompletely understood [5, 6]. Increasing evidence indicates that neuroinflammatory processes play a contributory role in mood disorders [7].

Neuroinflammation may originate not only from central nervous system mechanisms but also from peripheral sources, particularly the gut. The gut plays a key role in neuromodulation and in regulating immune responses, thereby influencing intestinal, systemic, and neuroinflammatory processes [8]. Within this context, the microbiota–gut–brain (MGB) axis has gained increasing attention in the pathophysiology of mood disorders, with intestinal barrier dysfunction (“leaky gut”) recognized as a major driver of inflammation [9].

The intestinal epithelium forms a barrier that prevents luminal antigens, microbial products, and pathogens from entering the systemic circulation [10]. Disruption of the intestinal barrier allows bacterial components such as lipopolysaccharide (LPS) to enter the circulation, which in turn may trigger the release of pro-inflammatory cytokines, including IL-1β, IL-6, and TNF-α [11]. These circulating cytokines can promote microglial activation and neuroinflammation, contributing to the pathophysiology of mood disorders [12]. Integrity of the epithelial barrier is sustained by intercellular junctional complexes, with tight junctions (TJs) playing a central role in this regulation [13].

Tight junctions are specialized multiprotein complexes located at the apical junctions of epithelial cells, forming a dynamic barrier that regulates paracellular permeability [14]. Pro-inflammatory cytokines disrupt tight junctions and impair barrier function. As a result, permeability increases and microbial translocation occurs, which subsequently triggers systemic immune activation and cytokine release [15]. TJ integrity is regulated by various endogenous and exogenous factors, including pro-inflammatory cytokines, activation of Myosin Light Chain Kinase (MLCK), oxidative stress, and components of the gut microbiota, as well as structural proteins such as claudins, occludin, junctional adhesion molecules, and zonulin [16–18].

Among these regulators, zonulin was the first protein identified to regulate tight junctions and remains one of the most well-established biomarkers of intestinal permeability [19]. Zonulin is a human-derived protein that regulates epithelial and endothelial barrier function by modulating TJ permeability [20, 21]. When exposed to environmental triggers, intestinal epithelial cells secrete zonulin. This secretion leads to the breakdown of tight junctions. As a result, paracellular permeability increases, barrier integrity is compromised, and immune activation is initiated [19]. Zonulin, also known as pre-haptoglobin-2 (pre-Hp2), is the precursor of haptoglobin-2 and is encoded by the HP2 allele of the haptoglobin (HP) gene, which has two allelic variants: HP1 and HP2. Only the HP2 allele produces pre-Hp2, which functions as zonulin [21]. Through these mechanisms, elevated zonulin levels may mediate the transfer of peripheral inflammation to the central nervous system, thereby contributing to the pathophysiology of BD and MDD [22, 23]. This concept is consistent with the bidirectional nature of the gut–brain axis, and accumulating evidence supports the critical role of immune dysregulation and inflammation in the development of mood disorders [24].

In line with this, activation of the immune system has been observed during manic episodes [25]. Elevated levels of pro-inflammatory cytokines have also been reported in patients experiencing a manic episode compared with healthy controls [26]. Several studies further suggest that increased intestinal permeability is present in BD, indicating a possible role for the gut–brain axis [27].

The presence of elevated pro-inflammatory cytokines in patients with MDD supports its strong association with systemic inflammation [28]. Moreover, one study demonstrated that patients with depression, particularly those with chronic symptoms, had elevated IgA and IgM antibodies against LPS, which reflects immune activation and bacterial translocation [29]. Increased intestinal permeability has also been reported in MDD [30].

The present study aimed to investigate whether serum zonulin levels differ among patients with BD, MDD, and healthy controls, and to evaluate the potential role of zonulin as a clinical biomarker. Additionally, we examined whether the rs2070937 SNP in the HP gene is associated with serum zonulin levels and may contribute to the pathophysiology of these disorders.

## Methods

### Study design and setting

This case–control observational study was conducted between July 2023 and May 2024 at the Department of Psychiatry, Balıkesir University Faculty of Medicine, Turkey. The study protocol was approved by the Local Ethics Committee of Balıkesir University Faculty of Medicine and conducted in accordance with the principles of the Declaration of Helsinki. Written informed consent was obtained from all participants after they had been provided with detailed information about the study.

### Participants and clinical assessments

We initially enrolled 159 individuals. Following prespecified biomarker quality-control criteria, five participants (3.1%) were excluded listwise (ELISA: hemolysis/lipemia, BD *n* = 3, HC *n* = 1; rs2070937 genotyping QC failure, HC *n* = 1). The final analytic sample comprised 154 participants (MDD *n* = 56; BD *n* = 47; HC *n* = 51). The inclusion criteria were an age between 18 and 65 years, a diagnosis of BD type I or MDD according to the Diagnostic and Statistical Manual of Mental Disorders, Fifth Edition, Text Revision (DSM-5-TR), and provision of written informed consent. Eligibility was determined at screening before enrollment, during which a thorough medical history and medication review were obtained. Before diagnostic classification, all candidates were screened for lifetime DSM-5–threshold mania/hypomania and antidepressant-emergent mood elevation. Exclusion criteria comprised a current or lifetime organic mental disorder; schizophrenia, schizophreniform, or other psychotic disorders; pregnancy; substance use disorder; neurodegenerative disorders; current anxiety disorder; and any uncontrolled or severe medical condition. Individuals with coeliac disease, inflammatory bowel disease, other autoimmune disorders, or acute/chronic gastrointestinal inflammatory conditions, as well as those currently receiving systemic corticosteroids or other immunomodulatory therapies, were excluded.

All participants underwent the Structured Clinical Interview for DSM Disorders (SCID) to confirm diagnoses and to exclude comorbid psychiatric conditions. After the diagnostic evaluation, each participant completed a sociodemographic form documenting age, sex, smoking status, alcohol consumption, body mass index (BMI), chronic medical conditions, income level, and medication use. In patients with BD, manic symptom severity was assessed with the Young Mania Rating Scale (YMRS), an 11-item, clinician-rated instrument indexing the preceding 48 h; four items are scored 0–8 and seven 0–4, yielding a total of 0–60 (higher scores indicate greater severity) [31]. In the Turkish validation, internal consistency was Cronbach’s α ≈ 0.79; item–total correlations ranged 0.41–0.85, and inter-rater agreement across items (weighted κ) ranged 0.11–0.85 [32]. In patients with MDD, depressive symptoms were measured with the Hamilton Depression Rating Scale (HAM-D-17), a clinician-rated instrument using mixed 0–2 and 0–4 anchors (total 0–52) [33]; the Turkish validation reported Cronbach’s α ≈ 0.75, split-half reliability ≈ 0.76, test–retest reliability *r* ≈ 0.85 over 5 days, and inter-rater reliability *r* ≈ 0.87–0.98 [34].

### Serum zonulin measurement

Venous blood samples were collected from all participants after 8–12 h of fasting into yellow-capped (biochemistry) tubes. Samples from yellow-capped tubes were centrifuged at 1500 × g for 10 min, and serum samples were separated, transferred into Eppendorf tubes, and stored at − 40 °C until analysis. Before analysis, the samples were thawed gradually, first at + 4 °C and subsequently at room temperature. Serum zonulin concentrations were measured in duplicate using a commercially available human zonulin ELISA kit (Elabscience, Houston, TX, USA) according to the manufacturer’s instructions. The mean of the duplicate measurements was used for analysis.

### Genotyping

Venous blood samples were collected into tubes containing EDTA for DNA extraction. Genomic DNA was isolated using a Genomic DNA Purification Kit (Thermo Fisher Scientific Inc., Massachusetts, USA) according to the manufacturer’s protocol.

The single-nucleotide polymorphism (SNP) rs2070937 (A > G) in the HP gene, located on chromosome 16 (position 72,055,841; GRCh38), was selected based on its reported associations with inflammatory and immune-mediated disorders, as well as psychiatric conditions such as schizophrenia, and its potential relevance.

Genotyping was performed using TaqMan allelic discrimination assays with predesigned probes (VIC for the wild-type allele and FAM for the variant allele) on a real-time PCR system (Applied Biosystems 7500 Real-Time PCR, California, USA). The PCR protocol consisted of an initial denaturation at 95 °C for 10 min, followed by 40 cycles of 95 °C for 15 s and 60 °C for 1 min. Negative controls and duplicate samples were included to ensure the reliability of the results. Genotype calls were analyzed using Applied Biosystems 7500 Real-Time PCR Software v1.5.1 (Applied Biosystems, Foster City, CA, USA). The context sequence for the rs2070937 polymorphism was ATTGCCAATGTACTTTCCTGAATGC[A/G]GCCAGAAACTGAGCCCACCCCTCCA.

### Statistical analysis

An a priori power analysis was conducted using G*Power version 3.1.9.4. For a one-way fixed-effects ANOVA with three independent groups (BD, MDD, and HC), assuming a medium effect size (f = 0.25), an alpha level of 0.05, and a desired power of 0.80, the required minimum total sample size was calculated as 159 participants. The achieved sample size of 154 yielded an actual power of approximately 0.79 for detecting medium effect sizes.

Data analyses were performed using IBM SPSS Statistics, version 27.0. The distribution of continuous variables was assessed using visual methods (e.g., histograms) and the Kolmogorov–Smirnov test. For normally distributed variables, one-way ANOVA was applied, whereas the Kruskal–Wallis test was used for non-normally distributed variables.

Post-hoc group comparisons were conducted using Tukey’s test for normally distributed data and the Mann–Whitney U test for non-normally distributed data. In pairwise comparisons with the Mann–Whitney U test, Bonferroni correction was applied to adjust for multiple testing.

Categorical variables were analyzed using cross-tabulations and the Chi-square test. Comparisons between two independent groups with normally distributed variables were performed using Student’s t-test. Genotype distributions were assessed for Hardy–Weinberg equilibrium using the Chi-square (χ²) test within each group.

Receiver operating characteristic (ROC) curve analysis was conducted to evaluate the diagnostic performance of serum zonulin levels, and sensitivity and specificity values were calculated. Internal consistency of all inventories was assessed using Cronbach’s α.

## Results

Of 159 enrolled participants, five (3.1%) were excluded based on biomarker quality control (ELISA hemolysis/lipemia: BD *n* = 3, HC *n* = 1; genotyping failure: HC *n* = 1), yielding a final analytic *N* = 154 (96.9%). Within the analytic sample, there were no missing data across psychometric inventories, ELISA measurements, or rs2070937 genotypes.

**Fig. 1:**
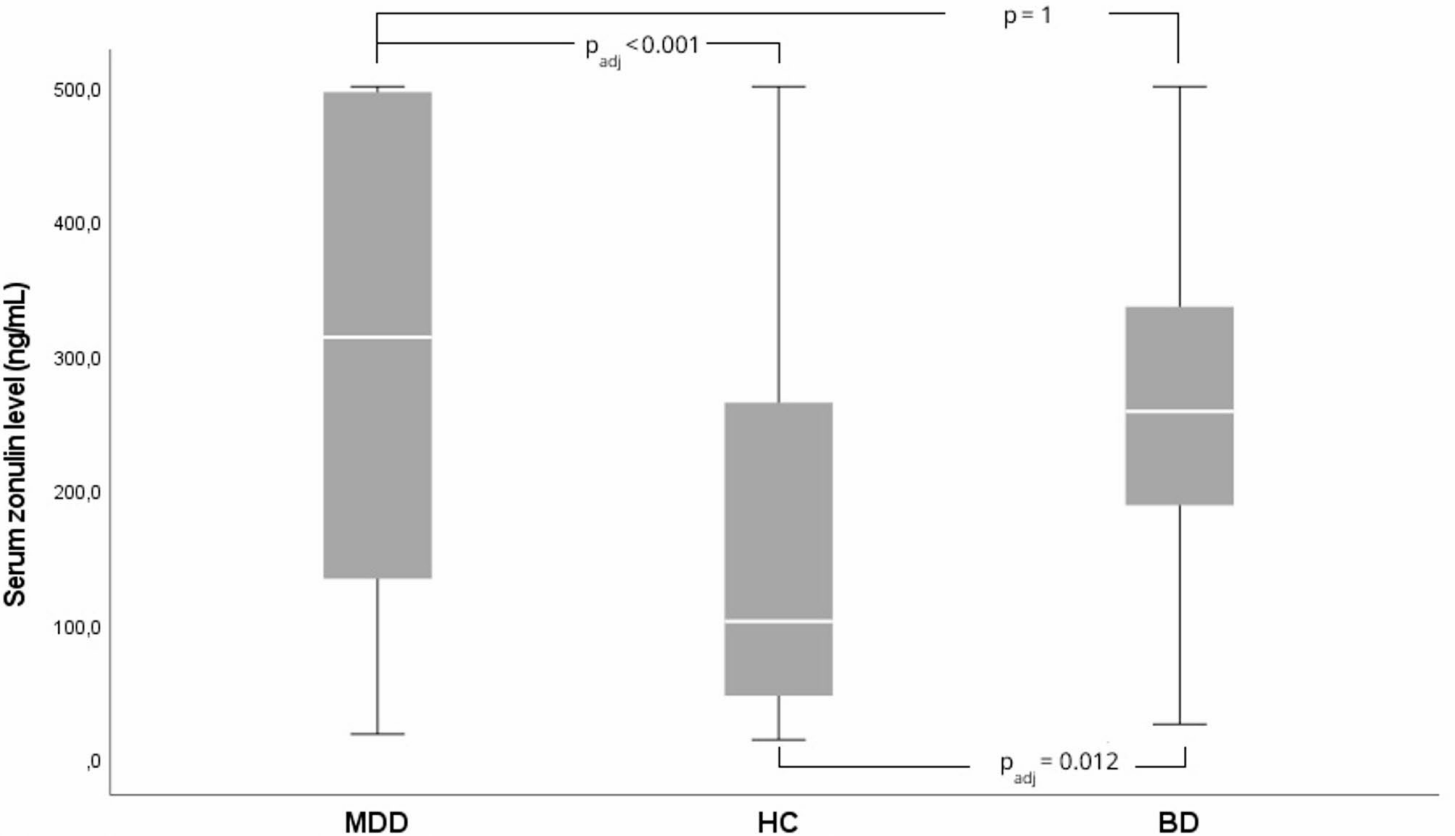
Box-plot of serum zonulin levels among patients with MDD, BD, and HC. The horizontal lines inside the boxes indicate median values; whiskers represent the minimum and maximum values. Zonulin levels were significantly higher in MDD compared to HC (*p*_*adj*_ < 0.001) and in BD compared to HC (*p*_*adj*_ = 0.012), whereas there was no significant difference between MDD and BD (*p* = 1.0)

The median (min–max) serum zonulin levels (ng/mL) were 258.00 (25.0–500) in the BD group, 313.19 (17.7–500) in the MDD group, and 101.65 (13.4–500) in the HC group. A significant overall difference was observed among the three groups (*p* < 0.001; Fig. 1). Post-hoc pairwise analyses showed that both the BD (*p*_*adj*_ = 0.012) and MDD (*p*_*adj*_ < 0.001) groups had significantly higher zonulin levels than controls, whereas BD and MDD did not differ from each other (*p* = 1.0; Table 1).

**Table 1:** Pairwise comparison of serum Zonulin levels between groups (p_adj_-values)

Groups	HC	MDD	BD
HC	1	< 0.001	0.012
MDD	-	1	1
BD	-	-	1

HC: Healthy controls; MDD: Major depressive disorder; BD: Bipolar disorder. Bonferroni correction was applied for multiple comparisons.

In terms of age distribution, mean ± SD values were 40.85 ± 13.25 years in the BD group, 42.3 ± 13.6 years in the MDD group, and 38.31 ± 12.01 years in the HC group, with no significant difference between groups (*p* = 0.277). Spearman’s correlation revealed no significant association between age and serum zonulin levels (*r*s = 0.152, *p* = 0.059; Table 2).

Analysis stratified by sex demonstrated significantly higher zonulin levels in females (276.75 [45.5–500] ng/mL) compared to males (127.19 [13.4–500] ng/mL; *p* < 0.001). When analyzed within diagnostic groups, significant differences were observed in both males (*p* = 0.003) and females (*p* = 0.013).

Sex distribution was similar across groups, with females accounting for 53.2% of the BD group, 54.9% of the HC group, and 58.9% of the MDD group (*p* = 0.832; Table 2).

The mean BMI was 25.7 ± 4.24 in the BD group, 26.8 ± 4.15 in the MDD group, and 26.1 ± 3.94 in the HC group, with no significant differences between groups (*p* = 0.434). Spearman’s correlation analysis revealed a weak positive association between serum zonulin levels and BMI (*r*s = 0.183, *p* = 0.023; Table 2).

**Table 2:** Comparison of serum Zonulin levels, age, sex, and BMI among healthy Control, MDD, and BD groups

Demographic and Clinical Characteristics	HC	MDD	BD	*p*
Serum zonulin level [ng/mL, median(min-max)]	101.65 (13.4–500)	313.19 (17.7–500)	258.00 (25.0-500)	< 0.001
Age (mean ± SD)	38.31 ± 12.01	42.3 ± 13.6	40.85 ± 13.25	0.277
Sex (% female)	54.9	58.9	53.2	0.832
BMI (kg/m², mean ± SD)	26.1 ± 3.94	26.8 ± 4.15	25.7 ± 4.24	0.434

HC: Healthy Controls, MDD: Major Depressive Disorder, BD: Bipolar Disorder, BMI: Body Mass Index

The chronic disease status did not differ significantly between groups (*p* = 0.233); 21 participants (13.6%) reported having at least one chronic condition.

Cigarette consumption (pack-years) did not differ significantly between groups (BD: 0 [0–25], MDD: 0 [0–26], HC: 0 [0–30]; *p* = 0.332). Serum zonulin levels showed no correlation with cigarette consumption (*ρ* = − 0.023, *p* = 0.775).

The rs2070937 genotype distribution was consistent with Hardy–Weinberg equilibrium in all groups (BD: χ² = 4.22, *p* = 0.121; MDD: χ² = 1.62, *p* = 0.444; HC: χ² = 0.73, *p* = 0.693). Median zonulin levels were 242.21 ng/mL for the AA genotype, 249.03 ng/mL for AG, and 302.19 ng/mL for GG. Although levels were highest in the GG genotype, the difference was not statistically significant (*p* = 0.622; Table 3).

**Table 3:** Genotypic distribution across study groups

Genotypes	HC	MDD	BD	*p*	𝑝
AA	22 (37.3%)	19 (32.2%)	18 (30.5%)	0.619*	0.678
AG	25 (30.1%)	31 (37.3%)	27 (32.5%)	0.679**	
GG	4 (33.3%)	6 (50%)	2 (16.7%)	0.476***	

* AA vs. AG + GG

** AG vs. AA + GG

*** GG vs. AA + AG

HC: Healthy Control, MDD: Major Depressive Disorder, BD: Bipolar Disorder. 𝑝: Spearman’s rank correlation coefficient

In the BD group, no significant correlation was found between serum zonulin levels and Young Mania Rating Scale scores (*r*s = − 0.086, *p* = 0.564). In the MDD group, no significant correlation was observed with the Hamilton Depression Rating Scale (*r*s = 0.235, *p* = 0.082). In the HC group, the correlation with HAM-D was likewise non-significant (*r*s = − 0.258, *p* = 0.068) (Table 4).

**Table 4:** Correlation between serum Zonulin levels and clinical scale scores across diagnostic groups

Groups	HAM-D	YMRS
MDD	rs = 0.235 *p* = 0.082	-
HC	rs = -0.258 *p* = 0.068	-
BD	-	rs = -0.086 *p* = 0.564

HC: Healthy control; MDD: Major depressive disorder; BD: Bipolar disorder; rs: Spearman’s rank correlation coefficient (rho); HAM-D: Hamilton Depression Rating Scale; YMRS: Young Mania Rating Scale

Analysis of suicidal ideation or behavior status showed that, in the BD group, median serum zonulin levels were 281.69 (69.8–500) ng/mL in individuals with suicidal ideation and 254.75 (25.0–500) ng/mL in those without (*p* = 0.749). In the MDD group, the corresponding values were 327.89 (17.7–500) ng/mL and 290.39 (17.7–500) ng/mL, with no significant difference observed (*p* = 0.873).

Internal consistency (Cronbach’s α) in our sample was HAM-D = 0.86 and YMRS = 0.81.

**Fig. 2:**
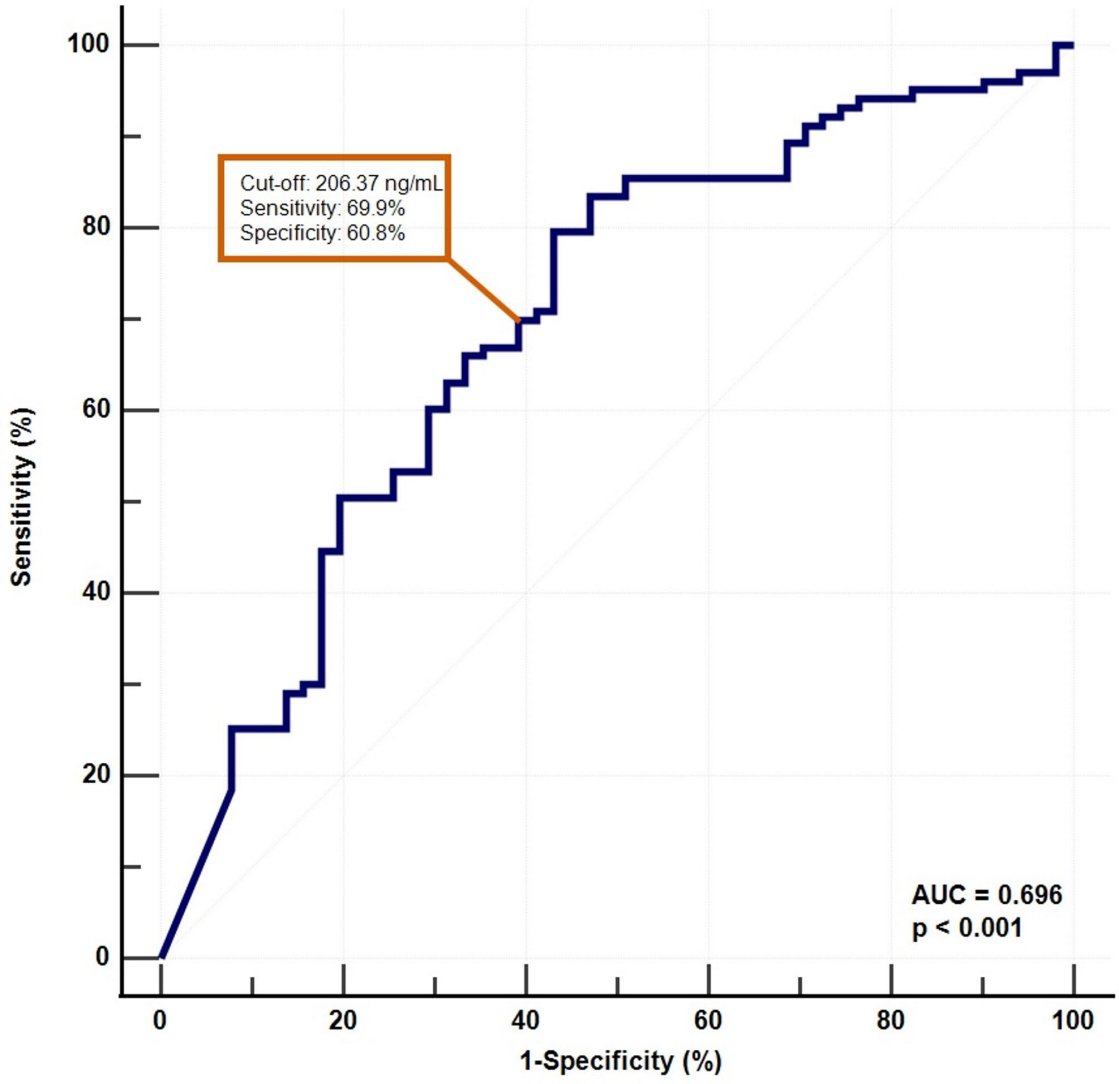
Receiver operating characteristic (ROC) curve showing the diagnostic ability of serum zonulin levels in differentiating patients with mood disorders (MDD and BD) from healthy controls. The optimal cut-off value was 206.37 ng/mL, with a sensitivity of 69.9% and a specificity of 60.8% (AUC = 0.696, 95% CI: 0.617–0.767, *p* < 0.001)

**Fig. 3:**
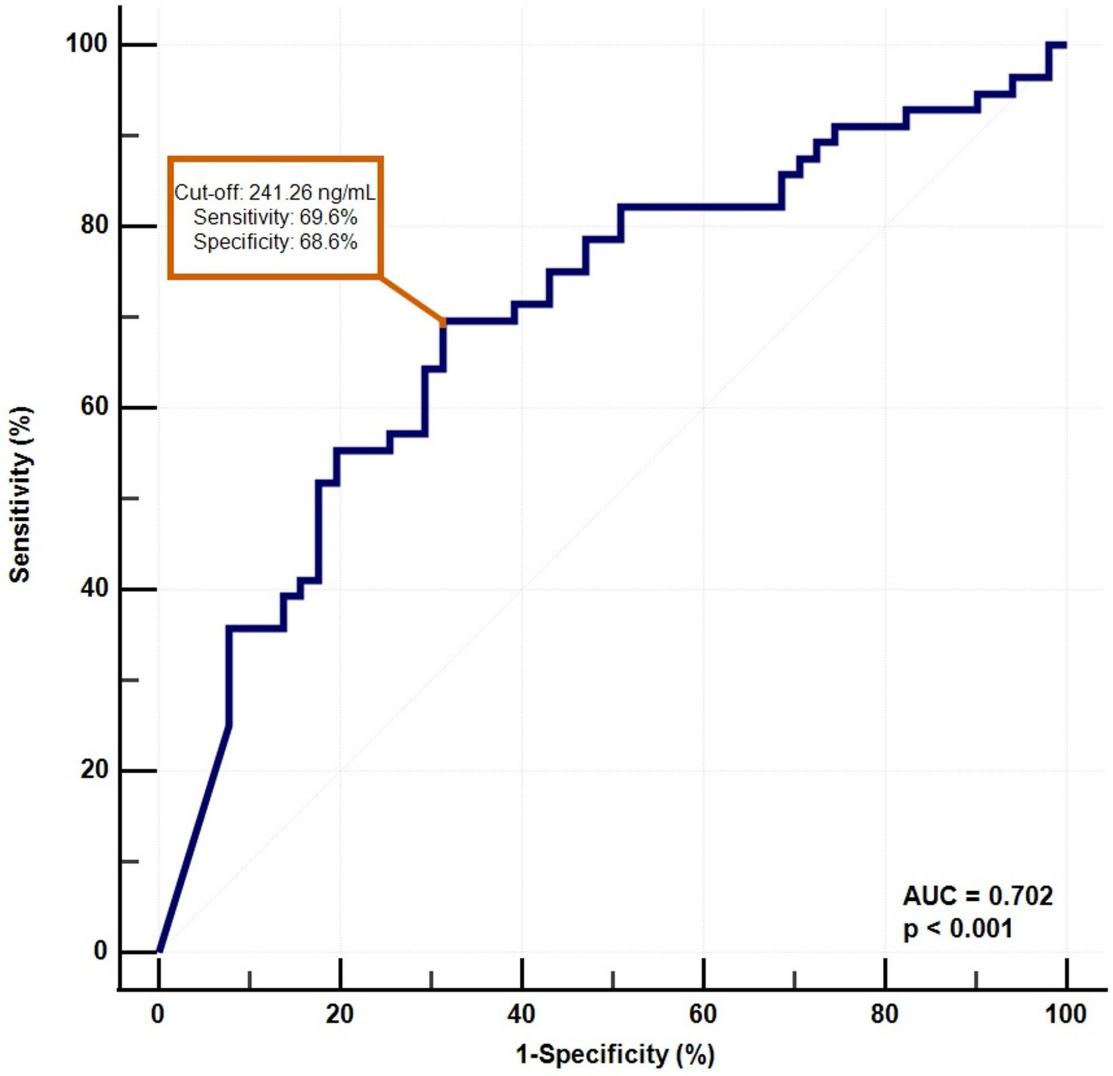
Receiver operating characteristic (ROC) curve showing the diagnostic ability of serum zonulin levels in differentiating patients with MDD from healthy controls. The optimal cut-off value was 241.26 ng/mL, with a sensitivity of 69.6% and a specificity of 68.6% (AUC = 0.702, 95% CI: 0.606–0.787, *p* < 0.001)

**Fig. 4:**
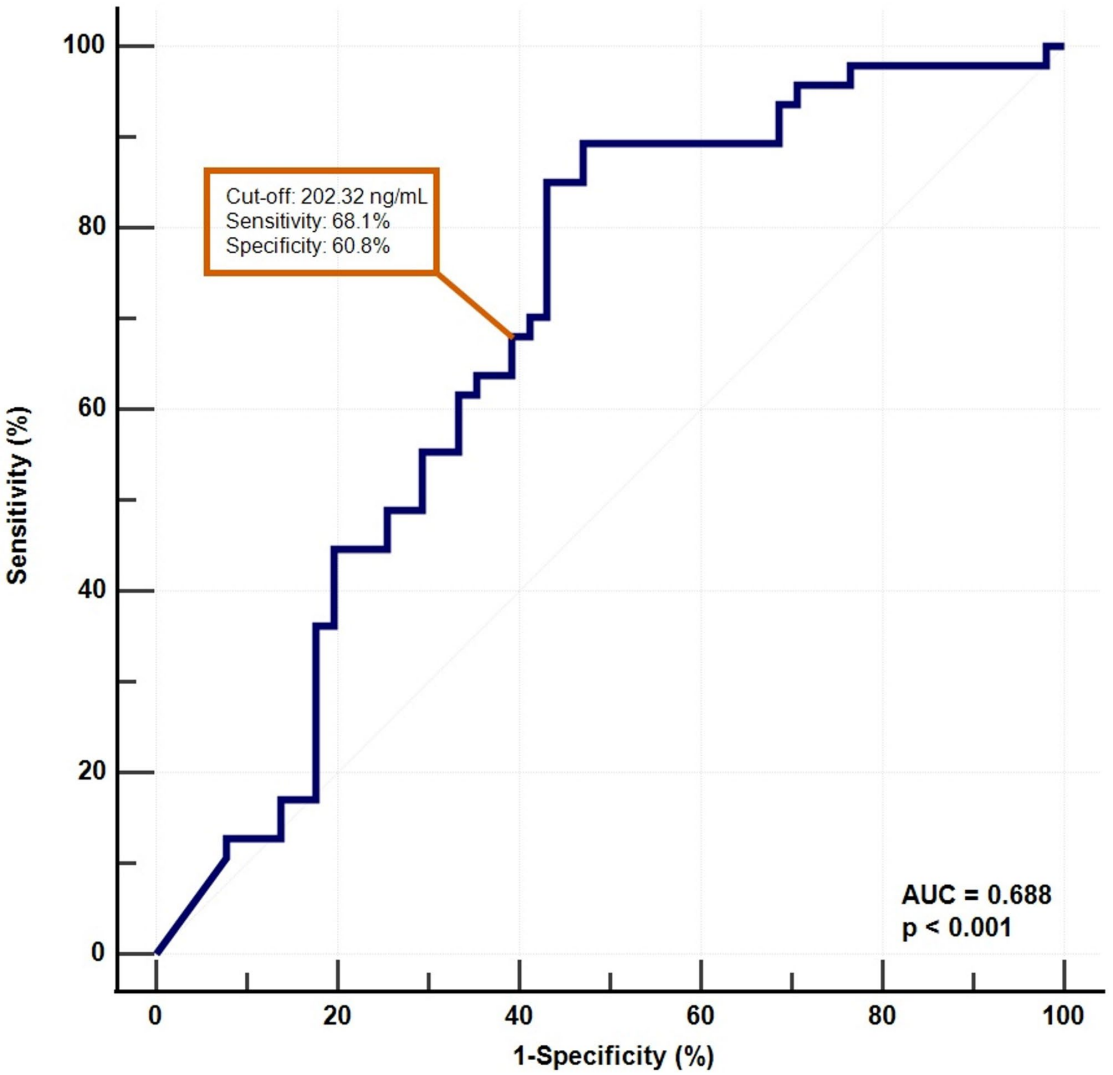
Receiver operating characteristic (ROC) curve showing the diagnostic ability of serum zonulin levels in differentiating patients with BD from healthy controls. The optimal cut-off value was 202.32 ng/mL, with a sensitivity of 68.1% and a specificity of 60.8% (AUC = 0.688, 95% CI: 0.587–0.778, *p* < 0.001)

Finally, ROC analysis was performed to evaluate the diagnostic performance of serum zonulin levels. For patients with mood disorders, the AUC was 0.696 (95% CI: 0.617–0.767). At a cut-off value of 206.37 ng/mL, sensitivity and specificity were 69.9% and 60.8%, respectively (Fig. 2). In the MDD group, the AUC was 0.702 (95% CI: 0.606–0.787). Using a cut-off value of 241.26 ng/mL, sensitivity and specificity were 69.6% and 68.6%, respectively (Fig. 3). In the BD group, the AUC was 0.688 (95% CI: 0.587–0.778), with a cut-off of 202.32 ng/mL yielding 68.1% sensitivity and 60.8% specificity (Fig. 4).

## Discussion

Tight junctions are dynamic structures that play a central role in regulating intestinal epithelial permeability [35]. Tight junction regulation is crucial for maintaining barrier homeostasis [19]. Zonulin, an endogenous protein, functions not as a structural component but as a dynamic modulator of intestinal tight junction permeability [20]. Increased zonulin activity enhances intestinal permeability and promotes the translocation of peripheral inflammatory signals into the central nervous system [36]. Preclinical studies support a mechanistic role for zonulin in intestinal permeability and subsequent inflammation. In IL-10 knockout mice, administration of the zonulin inhibitor AT-1001 markedly reduced permeability and ameliorated colitis [37]. This mechanism may underlie BD and MDD by linking gut–brain axis dysregulation with inflammation [38]. Consistent with this, our finding of elevated serum zonulin levels in both groups suggests that zonulin-mediated barrier dysfunction may represent a common mechanism underlying mood disorders.

In our study, serum zonulin levels were significantly elevated in patients with BD compared to healthy controls, consistent with previous reports [22, 39, 40]. In particular, a study by Kılıç et al. found increased levels of both zonulin and claudin-5 in BD, regardless of the disease phase [22]. Similarly, Zengil & Laloğlu reported elevated zonulin and occludin levels across manic, depressive, and euthymic states, with levels positively correlating with BMI [39]. In contrast, Aydın et al. did not observe significant differences in plasma zonulin levels between patients with BD and controls, nor any associations with clinical severity or treatment response [41]. Differences in study design and patient characteristics may explain variability in study findings. Clinical state at evaluation, medication exposure, and metabolic comorbidities are factors that could influence circulating zonulin levels [42–44].

In the MDD group, we also observed elevated serum zonulin concentrations compared with healthy controls. This finding is consistent with previous reports demonstrating elevated serum zonulin levels in patients with major depressive and anxiety disorders, both of which have been associated with increased intestinal permeability and gut dysbiosis [45]. Similarly, elevated zonulin levels have been reported in adolescents with MDD [46]. However, results across studies remain mixed, as one study reported no differences in serum zonulin between patients with MDD and controls, whereas another found an inverse correlation with depression severity [47, 48].

Previous studies have reported no significant associations between serum zonulin levels and YMRS scores, HAM-D scores, or illness duration [22, 49]. Our analysis similarly did not reveal significant correlations between serum zonulin levels and mood symptom severity or illness duration. Our findings indicate that higher zonulin levels could represent a biological susceptibility factor for mood disorders. However, it does not appear to fluctuate based on the severity of their symptoms or the duration of the disease. Supporting this notion, a recent study in patients with bipolar disorder found no significant differences in zonulin levels across manic, depressive, and euthymic states [39].

Serum zonulin levels did not differ significantly between patients with and without suicidal behavior in either the BD or MDD groups. This finding is consistent with prior reports in BD, which also found no differences according to suicide attempt history [22]. In contrast, lower zonulin concentrations were reported in psychiatric patients with recent suicide attempts or suicidal ideation compared with both non-suicidal MDD patients and healthy controls [47]. One might speculate that differences in patient characteristics and the timing of assessments relative to suicidal events contributed to these findings. Suicidality may involve biological processes such as stress responses [50], immune activation [51], and neuroendocrine alterations [52], all of which can influence zonulin release and thereby alter intestinal permeability [43, 50, 53]. In contrast, when suicidality is assessed retrospectively or during periods outside of acute crisis, such associations may be less apparent.

Across all groups, females exhibited higher serum zonulin levels, consistent with some reports suggesting sex-related differences [22, 49], although other studies found no sex difference [47, 54]. In women with polycystic ovary syndrome (PCOS), elevated zonulin levels have been described, with positive correlations to insulin resistance and menstrual disorder severity [55]. However, another study examining PCOS women without metabolic syndrome found no significant differences compared with healthy controls [56]. One possible explanation is that sex hormones modulate intestinal permeability. Experimental studies have demonstrated that estrogens and progesterone can regulate tight junction proteins and maintain gut barrier integrity [57, 58]. These hormonal effects, possibly interacting with metabolic disturbances, may contribute to sex-related differences in circulating zonulin. However, since some cohorts reported elevated zonulin levels in women even without metabolic comorbidity, the relationship is unlikely to be explained by hormonal or metabolic factors alone.

We found a weak but significant positive correlation between serum zonulin and BMI. This finding aligns with previous reports, including a study that identified BMI as an independent predictor of zonulin in adults [59]. Pediatric studies have reported higher zonulin levels in overweight and obese children, with positive correlations to BMI [60]. Increased body mass may contribute to barrier dysfunction through insulin resistance and systemic inflammation [59, 61].

We examined the rs2070937 single-nucleotide polymorphism (SNP) within the haptoglobin (HP) gene, which encodes pre-haptoglobin-2 (pre-Hp2), also known as zonulin [21]. Although structural HP1/HP2 polymorphisms have been examined in relation to zonulin, the impact of single-nucleotide variants within the HP gene remains largely unexplored [20]. Rs2070937 has previously been investigated in psychiatric contexts, with one study reporting a significant association with schizophrenia susceptibility in a Han Chinese population [62]. Considering the growing interest in the gut–brain axis and the role of zonulin in regulating the intestinal barrier and neuroinflammation, we hypothesized that rs2070937 might influence serum zonulin levels and contribute to the pathophysiology of affective disorders, such as bipolar disorder and major depressive disorder.

Although rs2070937 is located within the HP gene that encodes pre-haptoglobin-2, our study found no significant association between this variant and serum zonulin levels. Genotype distributions of rs2070937 were similar across bipolar disorder, major depressive disorder, and healthy controls, suggesting that this polymorphism does not influence either disease susceptibility or zonulin levels in these conditions. These findings imply that rs2070937 may lack functional relevance in modulating zonulin expression or activity, despite its genomic proximity to coding regions. Given the established link between zonulin and the HP2 structural allele, it is more plausible that zonulin production is determined by HP gene structure (HP1 vs. HP2) rather than by intronic or promoter variants. Although no significant associations were identified, these null findings contribute to clarifying the genetic mechanisms underlying gut permeability in psychiatric conditions.

Meta-analytic data indicate that 10–13% of patients initially diagnosed with MDD convert to BD within 10 years [63]. Therefore, some occult BD in our MDD arm cannot be entirely ruled out. Any such non-differential misclassification would be expected to bias between-group contrasts toward the null rather than produce spurious positives. Despite this, zonulin was significantly higher in both MDD and BD than in controls. Prospective, longitudinal studies directly comparing baseline zonulin between subsequent converters and non-converters are warranted to establish its prognostic validity for BD conversion.

## Strengths and limitations

To our knowledge, this is the first study to evaluate serum zonulin levels in patients with bipolar disorder, major depressive disorder, and healthy controls within a unified design. We also examined the HP rs2070937 polymorphism to assess whether it may modulate serum zonulin levels and contribute to susceptibility to mood disorders.

The cross-sectional design of this study limits the ability to draw causal inferences regarding the relationship between serum zonulin levels and bipolar disorder or major depressive disorder. Additionally, dietary habits that may influence serum zonulin levels were not assessed. Our design provided adequate sensitivity for medium effects but not for small effects; consequently, null findings for subtle contrasts should be viewed cautiously and revisited in larger studies.

## Conclusions

This study demonstrated that serum zonulin levels were higher in patients with BD and MDD compared with healthy controls. The results support a potential involvement of intestinal barrier dysfunction in the pathophysiology of mood disorders. Serum zonulin levels were not associated with illness duration, symptom severity, or suicidal behavior, suggesting that they may not directly reflect the clinical course of these disorders. No significant association was detected between the rs2070937 polymorphism of the HP gene and either serum zonulin levels or disease susceptibility. These findings add to the growing body of evidence linking gut permeability with affective disorders. Larger longitudinal studies are required to clarify causal relationships and potential therapeutic implications.

## Data Availability

The datasets used and/or analysed during the current study are available from the corresponding author on reasonable request.

## Abbreviations

BD: Bipolar Disorder
BMI: Body Mass Index
HC: Healthy Controls
HAM-D: Hamilton Depression Rating Scale
H: Haptoglobin
IL-10: Interleukin-10
IgA: Immunoglobulin A
IgM: Immunoglobulin M
LPS: Lipopolysaccharide
MDD: Major Depressive Disorder
MLCK: Myosin Light Chain Kinase
PCR: Polymerase Chain Reaction
pre-Hp2: Pre-haptoglobin 2
SNP: Single-Nucleotide Polymorphism
SCID: Structured Clinical Interview for DSM Disorders
TJs: Tight Junctions
TNF-α: Tumor Necrosis Factor-alpha
YMRS: Young Mania Rating Scale
